# Evolution of RXLR-Class Effectors in the Oomycete Plant Pathogen *Phytophthora ramorum*

**DOI:** 10.1371/journal.pone.0079347

**Published:** 2013-11-07

**Authors:** Erica M. Goss, Caroline M. Press, Niklaus J. Grünwald

**Affiliations:** Horticultural Crops Research Laboratory, Department of Agriculture Agricultural Research Service, Corvallis, Oregon, United States of America; University of Iceland, Iceland

## Abstract

*Phytophthora* plant pathogens contain many hundreds of effectors potentially involved in infection of host plants. Comparative genomic analyses have shown that these effectors evolve rapidly and have been subject to recent expansions. We examined the recent sequence evolution of RXLR-class effector gene families in the sudden oak death pathogen, *P. ramorum*. We found that *P. ramorum* RXLR effectors have taken multiple evolutionary paths, including loss or gain of repeated domains, recombination or gene conversion among paralogs, and selection on point mutations. Sequencing of homologs from two subfamilies in *P. ramorum*’s closest known relatives revealed repeated gene duplication and divergence since speciation with *P. lateralis*. One family showed strong signatures of recombination while the other family has evolved primarily by point mutation. Comparison of a small number of the hundreds of RXLR-class effectors across three clonal lineages of *P. ramorum* shows striking divergence in alleles among lineages, suggesting the potential for functional differences between lineages. Our results suggest future avenues for examination of rapidly evolving effectors in *P. ramorum*, including investigation of the functional and coevolutionary significance of the patterns of sequence evolution that we observed.

## Introduction

The interactions between plants and their pathogens are complex, encompassing several layers of defense and counter-defense by the plant host and multiple routes to pathogenicity and evasion of detection by the pathogen [1–3]. Plants have evolved mechanisms to detect the presence of pathogens via recognition of pathogen-associated molecular patterns (PAMPs), which result in a biochemical signaling cascade and eventually defense gene expression in the host. PAMP-triggered immunity (PTI), a general defense mechanism, restricts pathogen infection and growth. However, successful pathogens produce virulence factors to effectively circumvent or suppress PTI. Thus plant pathogens may secrete hundreds of proteins that act on plant targets, termed effectors, in order to colonize and reproduce in host plant tissues. The specific recognition of pathogens by host plants has been known for many years [4,5], but only relatively recently have researchers recognized the very large numbers of genes in eukaryotic plant pathogens that directly interact with plant host molecules [6–8]. Effectors are also thought to play a role in determining pathogen host range [9,10].

Whole genome sequencing of *Phytophthora* plant pathogens has revealed many hundreds of effectors [7,11,12]. *Phytophthora* is a genus of Oomycetes, which are fungus-like diploid eukaryotes that are more closely related to brown algae and diatoms than to Fungi [13]. The first three *Phytophthora* genome sequences published were for the soybean pathogen *P. sojae*, the sudden oak death pathogen *P. ramorum*, and the potato and tomato late blight pathogen *P. infestans* [7,11]. These pathogens have distinct life histories, which may be reflected in their complement of effectors. Both *P. sojae* and *P. infestans* have taxonomically narrow host ranges compared to *P. ramorum*, which is known to infect more than 100 hosts across diverse plant families [14]. *P. ramorum* attacks woody hosts, causing blight symptoms on stems and leaves as well as bleeding cankers on tree trunks, in contrast to *P. sojae*, which is soil-borne and causes root rot, and *P. infestans*, which causes blight on leaves, stems, and fruit. Comparisons among the distantly related *P. ramorum*, *P. sojae*, and *P. infestans* have highlighted the rapid divergence of putative effectors within the genus *Phytophthora*. These species show differences in genome structure and effector diversity. *P. infestans* has a large repetitive genome of 240 Mb, compared to 65 Mb for *P. ramorum* and 95 Mb for *P. sojae* [7,11]. While most core housekeeping genes in *P. infestans* have short intergenic regions containing few repetitive elements, its effectors tend to be surrounded by repetitive elements and have very long intergenic regions. This pattern was much less pronounced in *P. sojae* and even less in *P. ramorum*. The *P. infestans* genome appears highly dynamic with frequent duplication of genes in these regions of repetitive elements. *P. sojae*, in contrast, does not exhibit signs of recent expansion of effector gene families [15]. In *P. ramorum*, there is evidence of a more recent expansion of effector gene families compared to *P. sojae*.

The largest and most diverse class of effectors found in *Phytophthora* genomes contains all cloned avirulence (avr) genes [7,16–27]. Avirulence proteins are named as such because they trigger a rapid resistance response in host plants called effector-triggered immunity (ETI), but they also have virulence functions in susceptible host genotypes. These diverse effectors are therefore avirulence gene homologs (Avh) [28]. They are called RXLR-class effectors after the characteristic N-terminal motif found in most members of this group [7]. They are highly diverse in amino acid sequence and only a small number of orthologs have been found between sequenced genomes. They are modular in structure with a N-terminal signal peptide and ‘targeting’ domain, including the RXLR motif that is necessary for transport into host cells, and a C-terminal ‘functional’ or ‘effector’ domain [29–31]. Analyses of paralogs within *Phytophthora* genomes have identified signatures of positive selection in the C-terminal region of a number of RXLR-class effectors [11,28,32]. 

Compared to some bacterial plant pathogens, which may have around 30 effector genes, the several hundreds of effector genes in *Phytophthora* genomes are surprising. Why are there so many effectors? Researchers are primarily approaching this question using functional screens and by investigating the action of candidate effectors in plants. A complementary approach may be to investigate the evolution of recently duplicated effector genes, because it is repeated gene duplications over time that have led to dramatic expansion of effector gene families. Here, we examine the recent sequence evolution of paralogous RXLR-class effectors in *P. ramorum*.

*P. ramorum* is an exotic pathogen whose geographic origin is presently unknown [14]. Until recently, there were three clonal lineages named EU1, NA1, and NA2 according to the initial continent on which they were found and the order in which they were found [33,34]. These lineages have been estimated to be relatively old, such that they evolved in isolation prior to introduction to North America and Europe [35]. In 2011, a fourth diverged clonal lineage was discovered in the United Kingdom [36]. There is some evidence for phenotypic variation among lineages including differences in colony morphology, growth rates, and pathogenicity [37,38].

RXLR effectors in *P. ramorum* were previously divided into families based on amino acid sequence similarity [11,28]. Here, we further subdivided these families into paralogs that could be aligned based on DNA sequence identity. We then investigated the recent evolution of *P. ramorum* RXLR effector paralogs, specifically examining the relative roles of point mutations, recombination, and domain insertions/deletions. We examined two of our subfamilies in detail by sequencing the genes in the three *P. ramorum* clonal lineages and sister species. This allowed investigation of evolution of RXLR effectors since speciation of *P. ramorum* from the common ancestor of its close relatives, including inference of the chronology of gene family expansion and the nature of divergence among paralogs. These analyses go beyond previous bioinformatic screens for positive selection and suggest future avenues for examination of rapidly evolving effectors in *P. ramorum* and other plant pathogens exhibiting gene family expansion.

## Materials and Methods

### Identification of *P*. *ramorum* RXLR-class effectors

 A list of identified RXLR effector proteins was kindly provided by Rays Jiang and Brett Tyler [7,28]. Coding sequences were obtained for each gene from the *P. ramorum* genome sequence and subjected to multiple rounds of alignment using MUSCLE [39] to identify closely related sequences. Alignments were manually adjusted in BioEdit [40].

### Sequencing of homologs

*Phytophthora* isolates used in this work were stored in water on hemp seed at 20°C and maintained on cleared V8 agar prior to DNA extraction. Isolates were handled following the standard operating procedures associated with corresponding USDA APHIS permits and an exemption from the Director of the Oregon Department of Agriculture for work with *P. ramorum* under containment conditions. DNA was extracted from mycelia grown in V8 broth using the FastDNA SPIN kit (MP Biomedicals, Santa Ana, CA) using the protocol for yeast and fungi with minor modifications.

Primers for RXLR genes were designed using the Primer3 software [41]. To amplify and sequence the gene and surrounding flanking sequence from the three *P. ramorum* clonal lineages, primers were placed in the flanking regions such that they would be specific to a single gene, usually 100 to 300 bp upstream from the start codon and downstream from the stop codon. *PrAvh192* and *PrAvh169* were identical in gene and available flanking sequence and therefore could not be distinguished. These sequences are referred to as *PrAvh169*. To amplify and sequence orthologs from *P. lateralis* isolate 440, *P. hibernalis* isolate P3822, and *P. foliorum* isolate LT192, primers were placed inside each gene, such that primers were conserved among the *P. ramorum* lineages and in some cases conserved among genes. Once a portion of the gene was amplified, TAIL PCR was used to obtain the ends of the gene and flanking sequence. TAIL PCR uses a series of three reactions primed with specific nested primers internal to the known sequence paired with arbitrary primers [42]. When more than one amplified fragment was observed by gel electrophoresis after the third PCR, bands were separated by gel extraction (Qiagen, Valencia, CA). Once flanking sequence was obtained using TAIL PCR, specific primers were designed to amplify and sequence the entire gene. When more than one locus was amplified using a given pair of primers, PCR products were cloned using TOPO TA (Invitrogen, Life Technologies, Grand Island, NY) or pGem-T (Promega, Madison, WI) cloning kits. Primers for full genes are given in File S1; internal primers for each gene and specific TAIL PCR primers are available upon request. PCR was conducted using GenScript Taq (GenScript, Piscataway, NJ) using a final concentration of 2mM dNTPs and 2 to 3 mM MgCl_2_. The general PCR program consisted of initial denaturation at 94°C for 3 min, 35 cycles of 94°C for 1 min, primer annealing for 1 min at the primer-specific temperature (Table S1), and extension at 72°C for 1 min, with a final extension at 72°C for 10 min. PCR products were cleaned up prior to sequencing using ExoSAP-IT (USB, Cleveland, OH). PCR products or cloned fragments were sequenced on both strands on an ABI 3730 capillary sequencer using Big Dye Terminator 3.1 (Applied Biosystems, Life Technologies, Grand Island, NY) using the PCR primers, except for the TAIL PCR products, which were sequenced using only the third specific primer. Previous work indicated that there is little to no variation in coding sequences among isolates within the *P. ramorum* clonal lineages [35], therefore only 1 to 2 isolates per lineage were sequenced for most genes and results focus on the variation among lineages. All sequences generated have been deposited in NCBI GenBank under accession numbers EU850875-EU850938 and KF273331–KF273379 (Table S1).

### Identification of WY-domain

**Figure 1:**
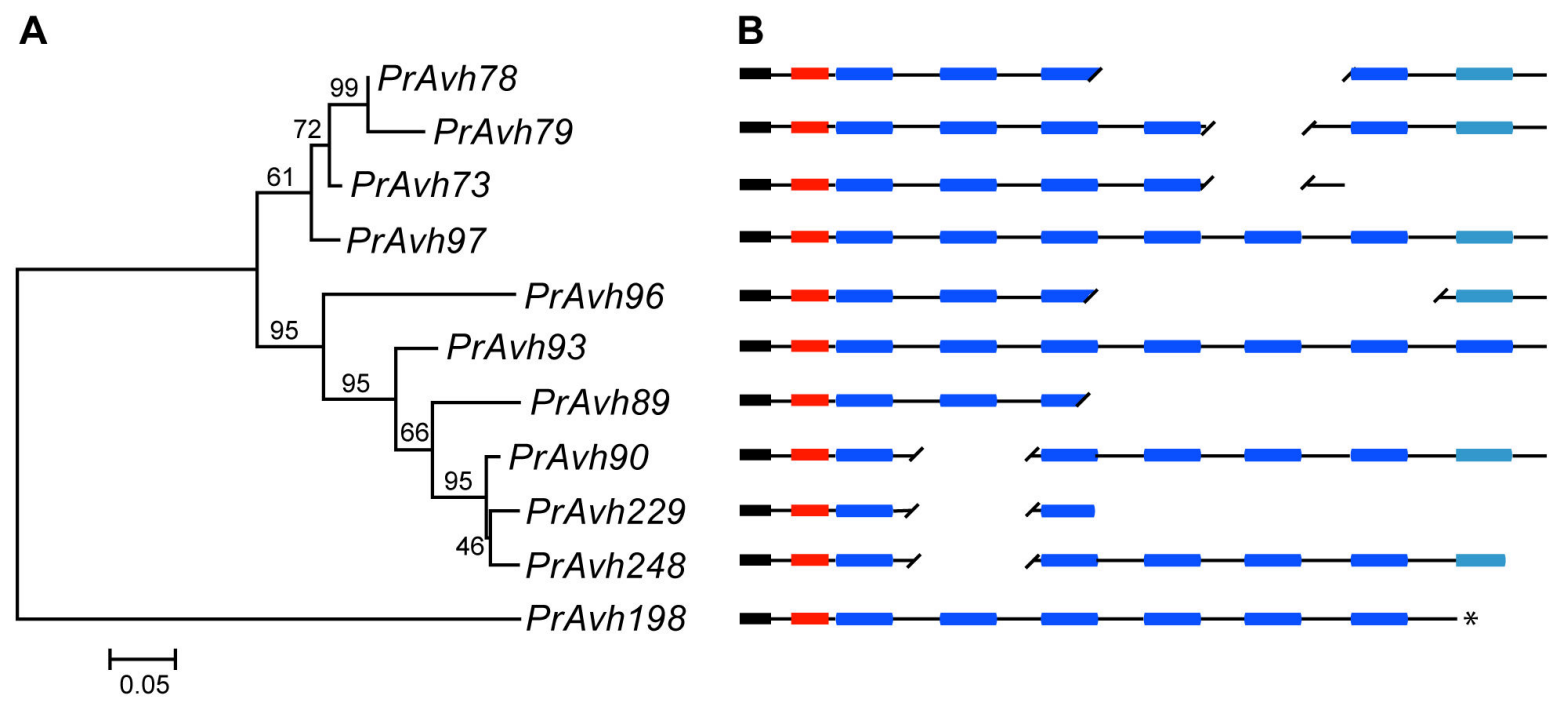
*P. ramorum* RXLR effector family with insertion/deletion of domains. Evolution in the domain structure of effectors is evident by comparing the phylogeny of the genes to their composition. A. The ancestral relationships among the genes inferred from a maximum likelihood tree of the targeting region (signal peptide through RXLR-DEER) with indels removed. Branch support is shown as a percentage of 500 bootstrap samples. The branch lengths are drawn to scale and measured in the number of substitutions per site. B. Schematic showing the aligned domain structure of the genes (not to scale). Gaps in the alignment are indicated using forward slashes. Colors of domains are: black – signal peptide, red – RXLR-DEER, and blue –WY-domain. WY-domains in lighter shade indicate low scoring matches to the HMM. *PrAvh198 was at the end of a scaffold and is missing the 3’ end of the gene.

**Figure 2:**
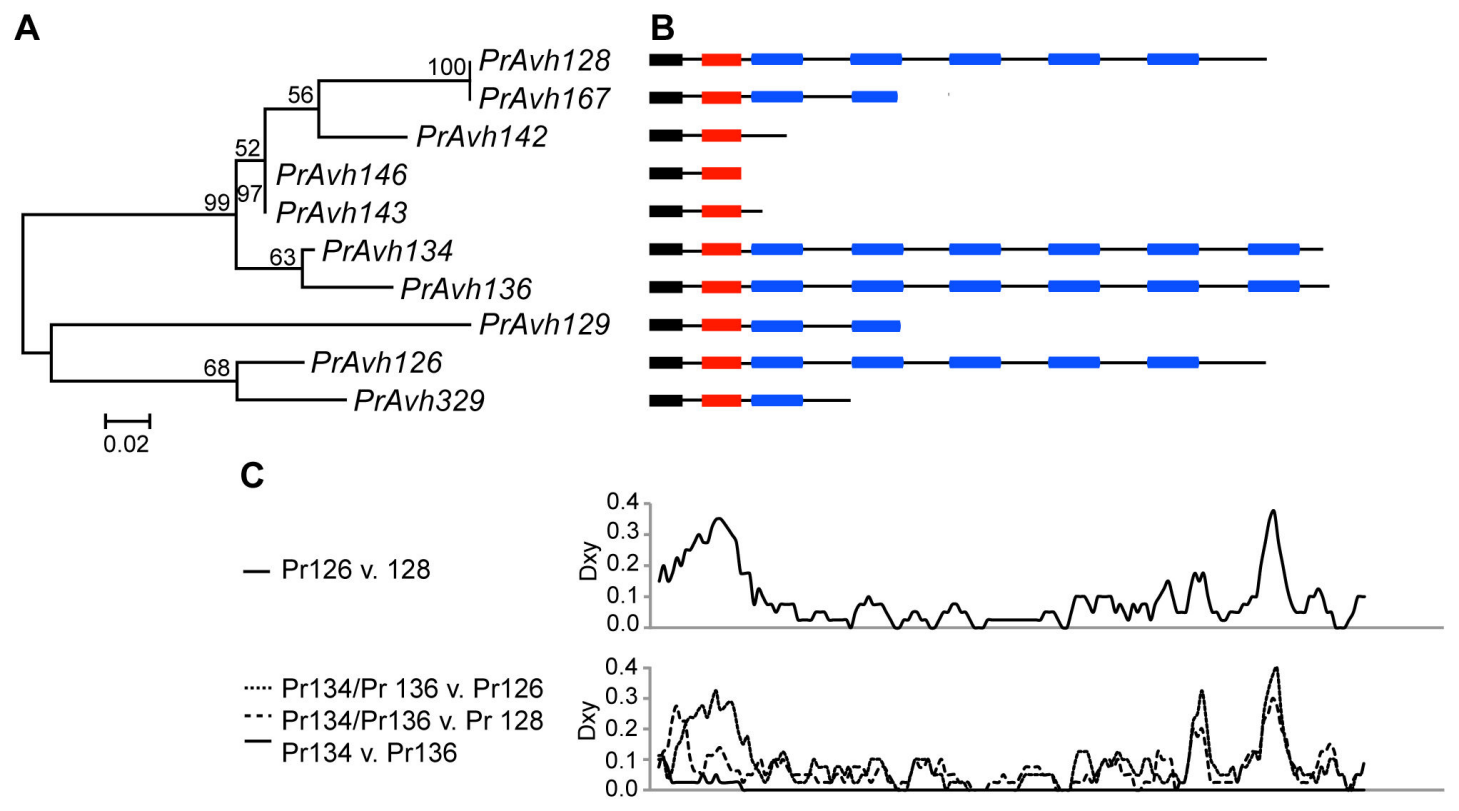
*P. ramorum* RXLR effector family with length polymorphism. Similar domain structures are found in different subclades and stop codons have shortened coding regions. A. Maximum likelihood tree of the targeting region (signal peptide through RXLR-DEER) with indels removed. Branch support is shown as a percentage of 500 bootstrap samples. The branch lengths are drawn to scale and measured in the number of substitutions per site. B. Schematic showing the domain structure of the aligned genes (not to scale). Colors are as in Figure 1. C. Sliding window analysis of nucleotide divergence between selected genes that share similar domain structures showing that the middle sections of the genes are relatively conserved. Sliding window analysis of amino acid replacements per site (Ka) showed a very similar pattern. Total length of alignment was 1825 bp, 1614 bp after indels were removed.

WY-domain sequences from *P. ramorum*, *P. infestans*, and *P. sojae*, compiled by Boutemy et al. [43], were used to build an HMM using HMMER v3.1b1 (http://hmmer.org). The HMM was used to identify the WY-domains in the two groups depicted in Figures 1 and 2.

### Phylogenetic analysis

Multiple alignments were produced using ClustalW [44], as implemented in BioEdit 7.0.9 [40], or MUSCLE [39] if the ClustalW alignment appeared poor. Difficult alignments were performed using amino acid sequences and then back-translated to nucleotides. All phylogenetic analysis of nucleotide sequences used PhyML [45] as implemented in Geneious Pro 5.0 (Biomatters Ltd, Auckland, New Zealand) to infer maximum likelihood phylogenies. All data sets used the Tamura and Nei [46] (TN) nucleotide substitution model and 6 substitution rate categories. The transition/transversion ratio, proportion of invariable sites, and gamma distribution parameter were estimated from the data. Bootstrapping was conducted to assess branch support with 100 or 500 bootstrap samples. The neighbor-joining method was also used to infer trees for all data sets using the composite likelihood method for calculating genetic distances and 500 bootstrap samples. Maximum likelihood and neighbor-joining methods produced very similar results with the exception of short, weakly supported branches. One phylogenetic tree was inferred based on protein sequences and used the Minimum Evolution method [47] as implemented in MEGA4 [48]. Genetic distances were calculated using the JTT method [49]. Tree space was searched using the Close-Neighbor-Interchange algorithm at a search level of 1 from a starting tree generated by neighbor-joining. The pairwise deletion option was used to remove gaps only in pairwise comparisons. Five hundred bootstrap samples were used to assess branch support.

### Recombination

The program RDP3 beta41 [50] was used to infer recombination breakpoints in PrAvh gene subfamilies using the default settings.

### Positive selection

Variation in selection pressure among sites has been tested for paralogous genes and specifically among paralogous RXLR effectors in Oomycetes [28,32]. Selection in the evolutionary history of these genes can alternatively be examined by testing for variation in selection among lineages using branch-specific substitution models [51]. We used the program GABranch as implemented by www.datamonkey.org, which runs the HyPhy software package (HyPhy.org). Briefly, GABranch is a genetic algorithm that assigns branches to a fixed number of classes of ω, the ratio of rates of nonsynonymous (dN) to synonymous (dN) substitutions, and evaluates different models of rate variation among branches. Using this method, rate variation among specific branches does not have to be specified *a priori*. Model averaged probabilities of dN/dS > 1 are obtained using Akaike weights and results are not dependent on the selection of a single best model. The algorithm is computationally intensive, therefore the analysis was limited to *PrAvh205* paralogs and orthologs in *P. lateralis* and *P. hibernalis*. Prior to the analysis, these genes were tested for recombination (see above) and a non-recombinant fragment of the genes was identified for analysis with GABranch.

Several methods for detecting site-specific variation in selection are available. We used the fixed effects likelihood (FEL) and random effects likelihood (REL) methods [52–55], as implemented by www.datamonkey.org, and the M8a model, a REL method, in the program PAML 4.4 (http://abacus.gene.ucl.ac.uk/software/paml.html) to test for site-by-site variation in selection among *PrAvh205* paralogs and orthologs for the same region as used for the GABranch analysis. The model selection tool on www.datamonkey.org selected HKY as the best substitution model by AIC. This substitution model was used to run the FEL and REL analyses.

### Expression of RXLRs in planta

*P. ramorum* strain Pr-102 (Grünwald isolate #407, [7]) was grown on V8 agar (200 ml per liter clarified V8) incubated at 20 C for 7-10 days until plates were covered. Leaves were harvested from near the apex of *Rhododendron* cultivar Cat. Boursault plants, rinsed 3 times in water to remove particulates, and placed inverted on the surface of moist vermiculite in clear clamshell food packs. Leaves in the control treatment were inoculated with a sterile V8 agar plug with a scratch on the epidermis of the leaf directly below the agar plug. Leaves inoculated with Pr-102 were inoculated in the same way using plugs from 10-days old Pr-102 cultures. After inoculation, leaves were misted lightly with water and the lid was closed. Containers were incubated in natural light at room temperature and re-misted daily until the end of the experiment at 5 days post inoculation.

*Rhododendron* leaf tissue was harvested at 1, 2, 3, and 5 days post-inoculation (DPI), flash frozen in liquid N_2,_ and stored at -80 C. At least 3 leaves were sampled per timepoint. Leaf discs 1 cm in diameter were excised from leaf tissues centered over the inoculation point at 1, 2 and 3 dpi. At 1 and 2 dpi, there was some water soaking evident in tissues surrounding the inoculation point and at 3 dpi lesion formation was apparent. At 5 dpi, lesions were irregular in shape and 1-3 cm wide such that 5 mm-wide strips of leaf tissue were excised from immediately adjacent to the lesion margin. Frozen leaf tissue was ground using a mortar and pestle by repeatedly grinding and adding liquid N_2_ (minimum of five times) until a fine powder was obtained. Total RNA was extracted by adding 25 mg of ground leaf tissue to 900 μl of CTAB buffer (2% CTAB, 2.5% PVP-40, 2 M NaCl, 100 mM Tris-HCl pH 8.0, 25 mM EDTA pH 8.0, and 2% BME added immediately prior to use) and incubating at 65 C for 60 min with vortexing at 5 min intervals. After centrifugation, the samples were extracted twice with a chloroform/isoamyl alcohol mixture (24:1 v/v). Then, 500 μl of ethanol was added to the supernatant and the samples were processed with the RNeasy plant mini kit (Qiagen catalog num. 74904, Valencia, CA) following manufacturer’s directions for cleanup. On column DNase treatment was performed according to manufacturer’s directions (Qiagen catalog num. 79254). Samples were eluted in 30 μl water and RNA was concentrated using by ethanol precipitation, quantified using a spectrophotometer (Nanodrop, Wilmington, DE), and the concentration of all samples was adjusted to 1 μg/μl.

RNA controls were prepared for the RXLR effector genes *PrAvh36*, *PrAvh60*, *PrAvh68*, *PrAvh108*, *PrAvh120*, *PrAvh121*, *PrAvh205* and the plant gene cytochrome oxidase (COX) was used as an internal control [56]. Each gene was amplified using primers designed to the flanking regions of the gene and incorporated a T7 RNA polymerase (RNAP) binding site in the forward primer (5’ TAA TAC GAC TCA CTA TAG G) (File S1). The RNAP binding site served as a start point for generating RNA via RNA polymerase in an RNA expression vector (Megascript T7 kit, Life Technologies, Grand Island, NY). Primers were designed to amplify outside the open reading frame in order to include the entire transcript. The resulting PCR product size was close to 400 nucleotides. PCR conditions for all primer pairs were as follows: initial denaturation at 96 C for 3 min; 25 cycles of 96 C for 30 s, 55 C for 30 s, 72 C for 59 s; and a final extension at 72 C for 10 min. PCR products were purified using the MinElute PCR cleanup kit (Qiagen, Valencia, CA), quantified, and diluted to 200 ng/μl. The Megascript T7 kit was used to generate RNA from the purified and diluted PCR product according to the manufacturer’s protocol (Life Technologies, Grand Island, NY). RNA was treated with Turbo DNase and cleaned up using the MegaClear kit according to the manufacturer’s protocols (Life Technologies, Grand Island, NY). Samples were quantified and adjusted to 1 μg/μl for use in reverse transcription reactions. An aliquot of each sample was converted to cDNA and sequenced to confirm identity.

RNA transcripts in infected and control leaf samples were quantified using a two-step PCR protocol. SuperScript III (Invitrogen, Carlsbad, CA) was used for reverse transcription of RNA samples following the manufacturer’s protocol. Samples were quantified with a spectrophotometer and adjusted to the same concentration. Control cDNAs were serially diluted tenfold to generate a standard curve from 10 ag – 100 pg. Standard curves were prepared for each of the eight control cDNAs and these curves were used to quantify transcript copy numbers of the corresponding genes in leaf tissue samples. Transcript cDNAs were quantified using a Taqman quantitative PCR kit, Taqman primers and Fam-labeled probes (Life Technologies, Grand Island, NY). Master mix was prepared to a final reaction mixture of 3.5 mM MgCl, 200 μM dNTPs except 400 μM dUTP, 1X Taqman Buffer, 300 nM forward and reverse primers and 100 nM probe. Master mix was aliquoted to the reaction plate and 10 μl of cDNA was added to each reaction to minimize pipetting error. No-template negative control wells received 10 μl of water. Samples of cDNA were tested over a range of concentrations from 8 ng/μl, 80 ng/μl, to 800 μg/ul. Quantitative PCR was run on an 7500 Fast System (Applied Biosystems, Life Technologies, Grand Island, NY) using reaction conditions of 95 C for 10 min, followed by 45 cycles of 95 C for 3 s and 60 C for 30 s. The auto default threshold settings from the ABI 7500 Fast system were used to calculate C_T_ values.

Copy numbers of each transcript cDNA were calculated by absolute quantification using the standard curve for each corresponding gene. For statistical analysis, copy numbers were log transformed to normalize variance. Changes in gene expression across sampling times were evaluated using two-way analysis of variance with main factors gene and days post infection. Single factor analyses of variance were performed on the data for each gene separately, followed by Tukey’s HSD test, to examine significant changes in copy number across time points.

## Results and Discussion

In the *P. ramorum* genome sequence (isolate Pr-102, NA1 clonal lineage), there were approximately 70 *PrAvh* subfamilies made up of at least two genes whose nucleotide sequences could be aligned across most or all of the coding sequences. We used these subfamilies as starting points for examining the recent evolution of *P. ramorum* RXLR-class effectors. These subfamilies or groups of subfamilies make up Jiang et al.’s AGs [28] and Haas et al.’s RXLR families [11], both based on amino acid similarities. Thus, we effectively subdivided Jiang et al. AGs or Haas et al. families that exhibited poor alignment at the nucleotide level. Subfamily membership was determined by nucleotide sequence identity in the N-terminal and/or C-terminal coding regions of genes. We specifically looked for examples of domain swapping, in which genes shared high similarity with one subfamily in the N-terminal region but another subfamily in the C-terminal, but none were observed. The number of genes in subfamilies ranged from 2 to 12. More than half of the subfamilies had members with different stop codon positions and could include pseudogenes. Seven additional families had length polymorphisms due to indels. There were 29 subfamilies with only two members; three of these were composed of two sequences that were identical to each other and at least one of these three was likely to be an assembly error. We may also be missing genes that are not in the available assembly.

### Evolution by insertion/deletion of motifs

Approximately half of *Phytophthora* RXLR genes contain C-terminal WY-domains [28,43,57]. In effectors recently examined from *P. capsici* and *P. infestans* as well as *Hyaloperonospora arabidopsidis*, the domain was found to form a conserved structural fold comprising three α-helices [43,58]. The WY-domain is necessary for avirulence function in some *P. sojae* effector proteins [30] and can be repeated 10 or more times in the amino acid sequence of an RXLR gene [28].

Five *P. ramorum* subfamilies were found to have length variation and to contain the WY-domain or other shared motifs. We investigated the evolution of two of these subfamilies that specifically exhibited variation in the number of tandem repeats of the WY-domain among genes. These genes also have a motif between the WY-domains, called the L motif [28], which is not present in the effectors with published structures [43]. We compared gene structure to genealogy, based on phylogenetic analysis of nucleotide sequences of the N-terminal targeting region (signal peptide plus RXLR and DEER motifs) and alignment of full nucleotide sequences. The subfamily shown in Figure 1 has varying numbers of repeated domains and variation in the location of the stop codon. Deletions of WY-domains have occurred in different locations in the gene alignment over time (File S2). In a second subfamily with variation in the number of WY-domains, genes with similar domain structure are found in different subclades (Figure 2A,B; File S3). Several of the genes are highly truncated, including *PrAvh143* and *PrAvh146*, which have stop codons following the targeting region. Sliding window analysis of nucleotide divergence across genes with similar domain structure showed high divergence at the beginning and end of the genes, but conservation in the region of the first two to three WY-domains (Figure 2C). Sliding window analysis of amino acid replacements per site (K_a_) produced very similar patterns to those in Figure 2C. This result is consistent with Boutemy et al.’s [43] observations that polymorphic amino acids in the C-terminal region are limited to a small number of surface-exposed residues in the protein and that the N-terminal regions were not structured. 

### Evolution by recombination among paralogs

**Figure 3:**
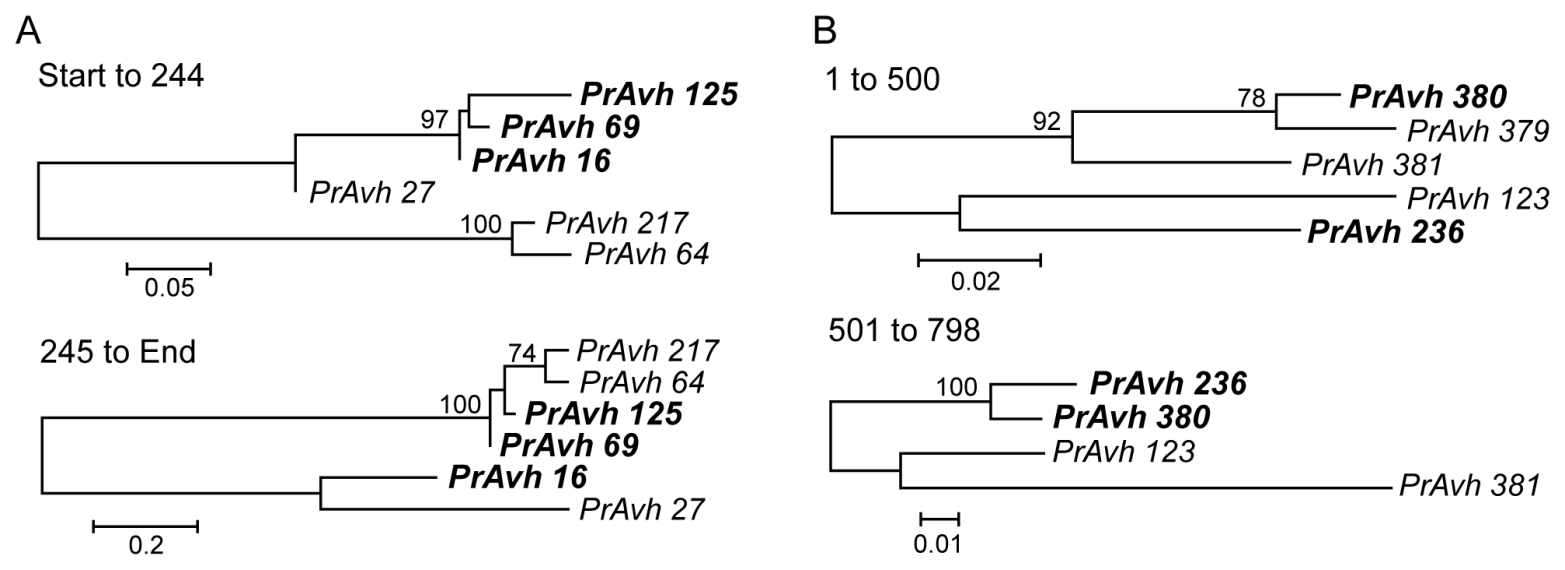
*P. ramorum* RXLR effector gene families exhibiting strong evidence of recombination. Changing evolutionary relationships in different regions of genes illustrate recombination among paralogs. Maximum likelihood trees were constructed for gene fragments on either side of estimated recombination breakpoints inferred using RDP. Bootstrap support for branches is out of 100 samples. The branch lengths are drawn to scale and measured in the number of substitutions per site. A. The phylogenetic relationships among genes in this gene family change between the N- and C-terminal coding regions of the gene. The recombination breakpoint is in the DEER motif. B. Recombination in the C-terminal functional region. *PrAvh379* has a frameshift mutation leading to a 507 bp truncated gene and thus is not included in the second tree.

 Recombination among effector gene paralogs has been reported in Crinkler (CRN) proteins in *Phytophthora infestans* [11] and in the bacterial plant pathogen *Pseudomonas syringae* [59]. We looked for signatures of recombination in *P. ramorum* RXLR-class effector gene subfamilies. Forty-eight subfamilies could be aligned and analyzed for recombination in the program RDP3. Out of these, 17 families produced at least weak evidence for recombination. However, some of these signals may have been due to high sequence divergence or poor alignment. Bootstrapped gene trees for segments defined by putative recombination break points were constructed, because strong bootstrap support for phylogenetic relationships that differ along segments of the gene is a clear indication of recombination or gene conversion among paralogs. Five subfamilies exhibited strong evidence of recombination or gene conversion among paralogs, as confirmed by bootstrapped trees. Two examples are given in Figure 3 and may represent especially recent recombination events between sequences and/or recombination between sequences sufficiently diverged to be detected.

**Figure 4:**
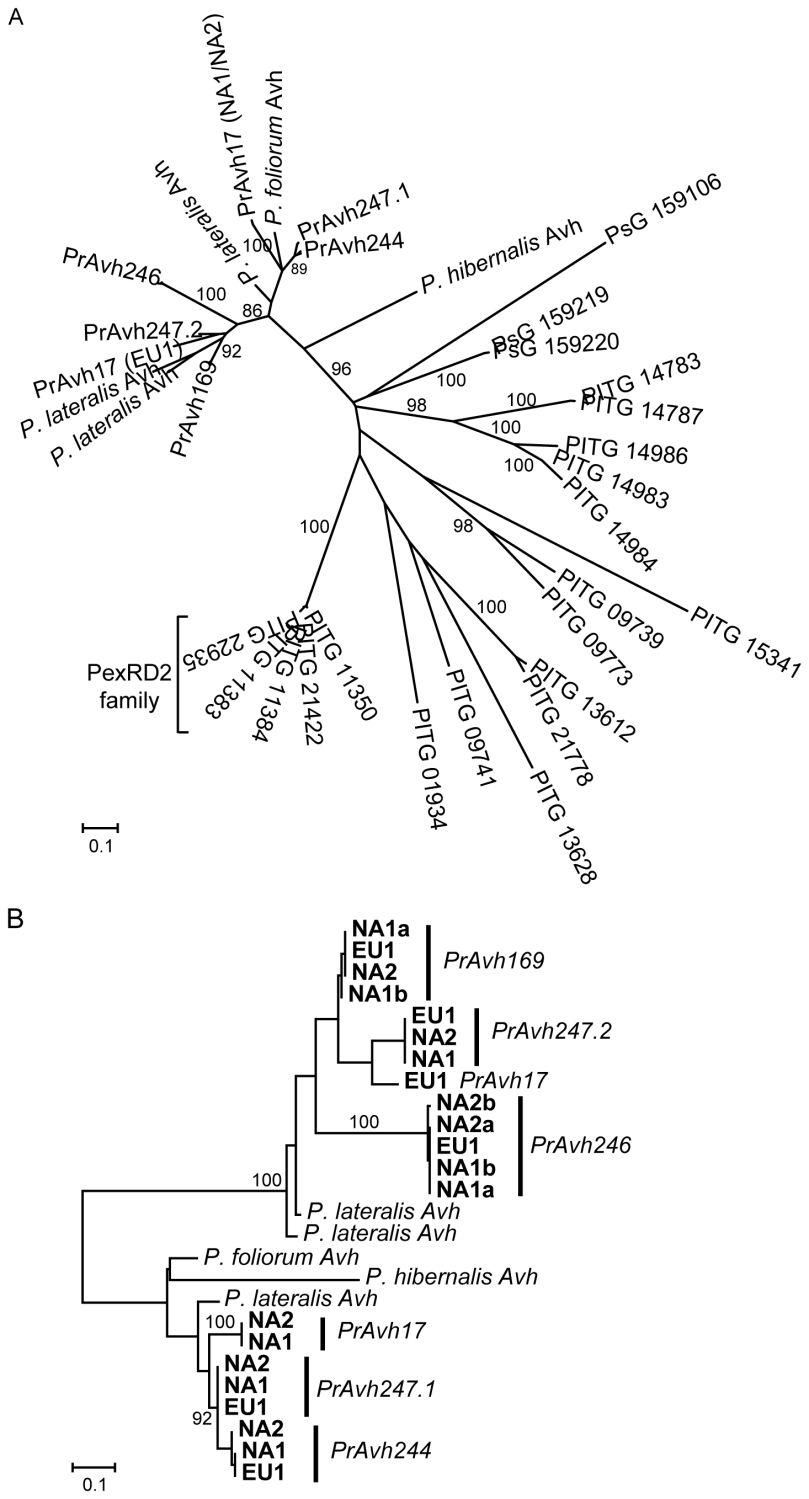
Relationships among orthologs and paralogs in an effector gene family with recombination. A family in *P. ramorum* that is orthologous to the PexRD2 family in *P. infestans* shows expansion and recombination in *P. ramorum*. A. Minimum evolution tree inferred using amino acid sequences for Haas et al.’s (2009) Fam6. This family includes the PexRD2 genes in *P. infestans*. Proteins from *P. infestans* have a PITG prefix and proteins from *P. sojae* a PsG prefix. Branch support is shown as a percentage of 500 bootstrap samples. The branch lengths are drawn to scale and measured in the number of substitutions per site. B. Maximum likelihood tree for nucleotide sequences representing the C-terminal region of Haas Fam6 effector genes from *P. ramorum*, *P. lateralis*, *P. hibernalis*, and *P. foliorum* indicating putative orthologous and paralogous relationships for this region. Branches with high bootstrap support, based on 500 bootstrap samples, are indicated. The branch lengths are drawn to scale and measured in the number of substitutions per site.

**Figure 5:**
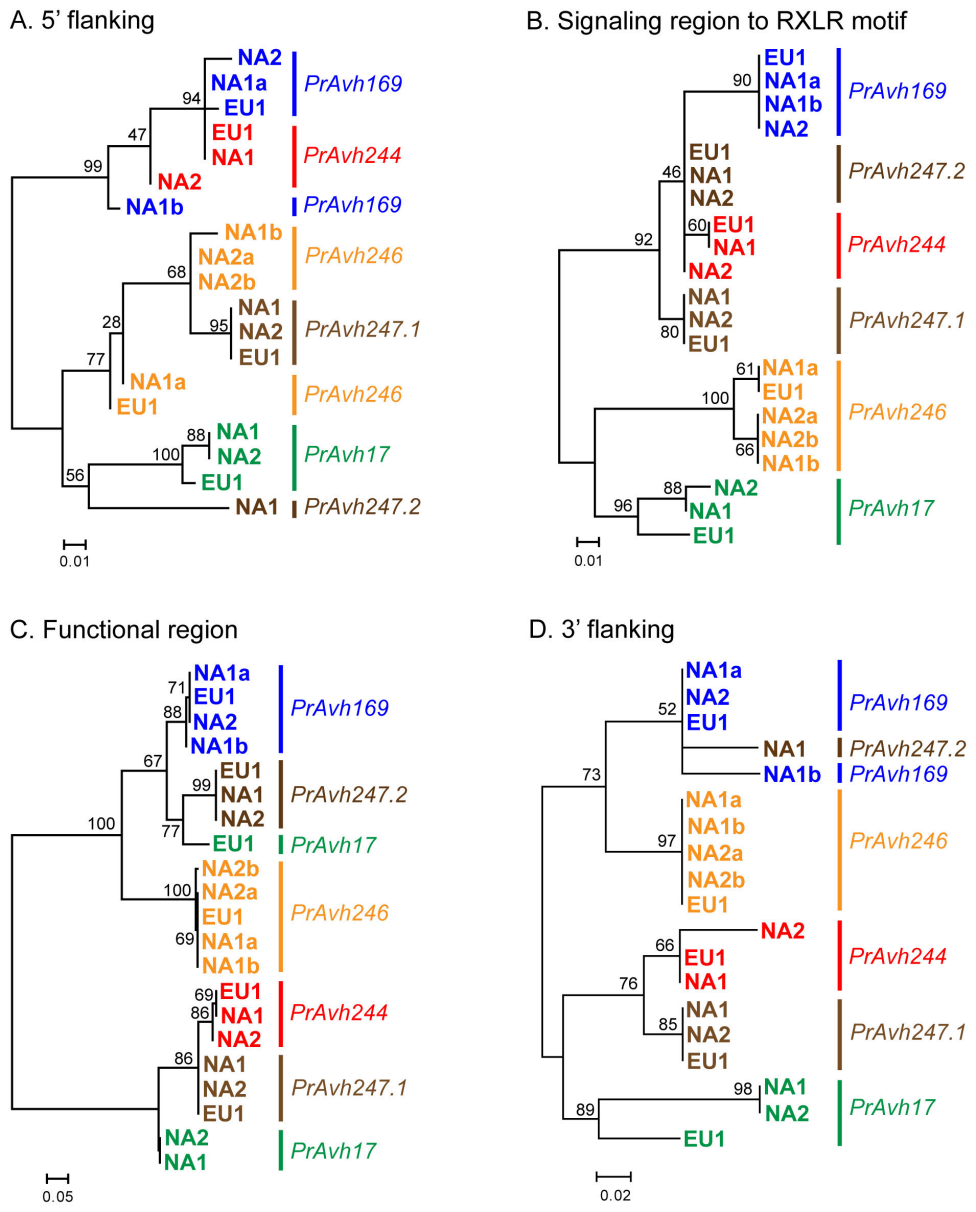
Recombination breakpoints occur between key gene regions in a *P. ramorum* RXLR effector family. Maximum likelihood trees were constructed for different fragments of genes exhibiting recombination among paralogs and show the changing evolutionary relationships among both genes and alleles between gene regions. Approximate recombination breakpoints were inferred using RDP and indels were removed. The sequences between inferred recombination breakpoints used for phylogenetic analysis were A. 5’ flanking sequences (201 bp), B. the targeting region (157 bp), from the start codon to RXLR motif, C. the functional (C-terminal) region (149 bp), and D. 3’ flanking sequences (70 bp). A short region around the DEER motif is not shown. Branch support is given as a percentage of 500 bootstrap samples. The branch lengths are drawn to scale and measured in the number of substitutions per site.

One subfamily with strong evidence of recombination represents *P. ramorum* homologs to the PexRD2 protein family in *P. infestans* (Haas et al. [11] family 6; Figure 4A). PexRD2 promotes cell death in *Nicotiana benthamiana* [21] and the crystal structure of the WY-domain was recently reported for this protein [43]. We sequenced each *P. ramorum* gene in this subfamily (PrFam6) in the NA1, NA2, and EU1 lineages to examine recombination and allelic variation (Table 1). *PrAvh169* and *PrAvh192* are identical and cannot be distinguished, thus we will only refer to *PrAvh169* from here on. Analysis of recombination using all *P. ramorum* alleles, including 5’ and 3’ flanking sequences, identified 4 putative breakpoints at the following locations: 1) just prior to the start of the coding region (ATG), 2) immediately downstream of the RXLR motif, 3) downstream of the EER motif, and 4) near the stop codon. Major changes in the phylogenetic relationships among genes were observed across breakpoints (Figure 5). For example, *PrAvh244* and *PrAvh169* share similar 5’ flanking sequences, but have dramatically different C-terminal and 3’ flanking sequences. In contrast, *PrAvh244* shares sequence similarity with *PrAvh247* except for the 5’ flanking sequence, for which *PrAvh247* and *PrAvh246* are similar. *PrAvh169*, *PrAvh244*, and *PrAvh246* are all located within a 36 kb region on *P. ramorum* scaffold 33 and *PrAvh247* is 170 kb from this group on the same scaffold. Allelic relationships are clear, with two exceptions. First, for *PrAvh17* the three *P. ramorum* lineages form a well-supported clade in the 5’ flanking and targeting regions, but the EU1 allele is highly diverged from the NA1 and NA2 alleles in the C-terminal, where it groups with the *PrAvh169*-*PrAvh246* clade (Figure 5A,B vs. C). This is likely to be the result of a recombination event between *PrAvh17* in EU1 and a gene in the *PrAvh169*-*PrAvh246* clade. Second, the primers designed to amplify *PrAvh247* amplified two distinct sequences from the NA1 lineage that are likely to be different loci rather than a single heterozygous locus, because they are diverged across much of the gene (*PrAvh247.1* and *PrAvh247.2* in Figure 5). These two different sequences were subsequently amplified from the NA2 and EU1 lineages as well. In the *P. ramorum* genome sequence, *PrAvh247* is a chimera of the two sequences, which appears to be an assembly error. The relationship of *PrAvh247.2* to the other genes changes across the alignment, resembling at times *PrAvh244* in NA2, *PrAvh17* in EU1, and *PrAvh169*.

**Table 1:** Nucleotide polymorphism in coding sequences of *P. ramorum PrAvh* genes.

Gene	Num. alleles	L^a^	S^b^	π^c^	π _syn_^c^	π _ns_^c^	Max K_a_/K_s_^d^	Recom^e^
*PrAvh17*	3	381	85	0.119	0.100	0.124	1.28*	Yes
*PrAvh169*	3	378	2	0.003	0	0.003	-*	
*PrAvh244*	2	378	5	0.007	0.018	0.004	0.21	
*PrAvh246*	3	375	4	0.006	0.007	0.005	0.58*	
*PrAvh247.1*	2	384	1	0.001	0	0.002	-*	
*PrAvh247.2*	1	378	0	0	-	-	-	
*PrAvh36*	1	336	0	0	-	-	-	
*PrAvh60*	5	375	25	0.023	0.037	0.019	0.66	Yes
*PrAvh68*	2	375	17	0.024	0.049	0.017	0.32	
*PrAvh108*	2	375	3	0.004	0.006	0.004	0.60	
*PrAvh120*	5	384	7	0.008	0.007	0.008	0.94*	
*PrAvh121*	5	384	35	0.039	0.063	0.033	0.89	Yes
*PrAvh205*	6	375	18	0.020	0.027	0.018	1.22*	

^a^Length after gaps removed. *PrAvh17* has a 3 bp indel between lineages and *PrAvh121* is heterozygous for a 12 bp indel in EU1 isolates.

^b^Number of segregating sites.

^c^Pairwise nucleotide polymorphism overall (π), at synonymous sites only (π _syn_), and nonsynonymous sites only (π _ns_).

^d^Maximum pairwise comparison of K_a_/K_s_ (ratio of nonsynonymous substitutions per site to synonymous substitutions per site). Asterisk indicates one or more pairwise comparisons with K_a_ > 0 and K_s_ = 0.

^e^Recombination detected within gene. Other genes may have experienced undetected recombination.

 We also attempted to sequence each gene from what are presently the three closest known relatives of *P. ramorum*: *P. lateralis*, *P. hibernalis*, and *P. foliorum*. Only one homolog was amplified from *P. hibernalis* and *P. foliorum*, however three alleles were amplified from *P. lateralis* (Figure 4). One of these is distinctly different from the other two and codes for a truncated protein due to a frameshift from a single base indel. This *P. lateralis* allele does not show dramatic divergence from its homologs in *P. ramorum*, suggesting that its potential pseudogene status is evolutionarily recent. The nucleotide sequence of the C-terminal region indicates that this gene is orthologous to the *P. hibernalis* and *P. foliorum* genes (Figure 4B). The other two *P. lateralis* sequences are allelic or a recent duplication. They are similar to each other in the C-terminal region, but across the entire coding sequence their divergence is greater than that observed between *P. ramorum* alleles (Figure 4B).

Our model for the evolution of this subfamily is that there was a single ancestral gene in the common ancestor of *P. hibernalis*, *P. lateralis*, and *P. ramorum* that duplicated prior to the *P. ramorum*-*P. lateralis* speciation event. Subsequent duplication in *P. ramorum*, and perhaps also in *P. lateralis*, led to the observed number of genes, which have experienced multiple recombination or gene conversion events. RXLR-class effectors in *P. ramorum* have therefore evolved by recombination between domains, as has been observed for other types of effectors [11,59]. Recombination events within major structural domains appear to be deleterious and removed by natural selection.

### Evolution by positive selection on point mutations

Analyses of RXLR-class effector gene evolution to date have focused on positive selection on point mutations that affect protein sequence via amino acid replacements. Jiang et al. and Win et al. showed that there was a significant signal of positive selection among paralogs in 28 *P. ramorum* subfamilies [28,32]. However, recombination can create a false signal of positive selection and we found that three of the 28 subfamilies have strong evidence of recombination or gene conversion. For subfamilies that appear to have evolved primarily by point mutation, we can test for evolution of effector proteins by positive selection.

**Figure 6:**
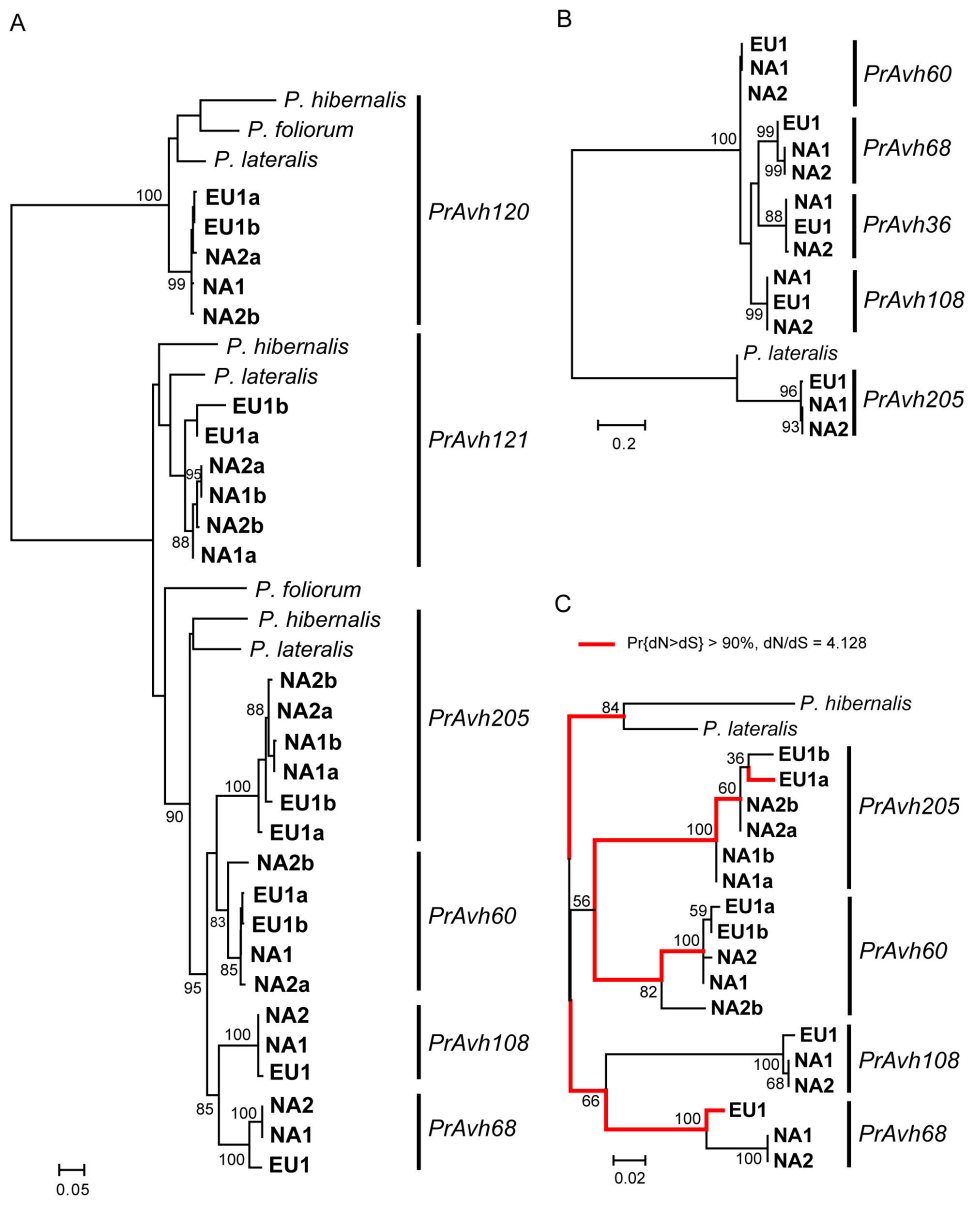
An RXLR effector gene family that has evolved primarily by point mutation. A. Maximum likelihood genealogy of the whole family and orthologous genes showing expansion of the family in *P. ramorum*. B. A maximum likelihood tree of the 5’ flanking region indicates orthologous and paralogous relationships among genes in the *PrAvh205* clade. C. Branches exhibiting a high probability of positive selection based on analysis with GAbranch for sites 130-330 in the C-terminal region of the genes. The model-averaged probability of dN>dS was greater than 90% for all red highlighted branches. All but the terminal *PrAvh68* branch for the EU1 lineage had probabilities greater than 95%. Branch support is indicated as a percentage of 500 bootstrap samples. The branch lengths are drawn to scale and measured in the number of substitutions per site.

We chose a subfamily with no apparent recombination in the *P. ramorum* genome sequence to resequence in the three clonal lineages (Table 1) and *P. ramorum*’s closest relatives. Three homologs could be amplified from *P. lateralis* and *P. hibernalis*, while only 2 were amplified from *P. foliorum*. The grouping of genes revealed by phylogenetic analysis of nucleotide sequences from all four species suggests that there were two or possibly three genes ancestral to this group (Figure 6A). *PrAvh120* has orthologs in all four species and is significantly diverged from the other genes in this subfamily. *PrAvh121* has orthologs in *P. hibernalis* and *P. lateralis*, while *PrAvh205*, *PrAvh60*, *PrAvh108*, and *PrAvh68* have a single homolog in *P. hibernalis* and *P. lateralis* suggesting that there were a series of duplications after speciation of *P. ramorum*. Similarity in 5’ flanking sequences between *PrAvh205* and the *P. lateralis* homolog indicates that *PrAvh205* is the orthologous gene and that *PrAvh60*, *PrAvh68*, and *PrAvh108* are paralogs of *PrAvh205* (Figure 6B). Interestingly, we could not amplify an ortholog to *PrAvh205* in *P. foliorum*, rather we amplified a gene with *PrAvh121* internal primers that is diverged from the other *PrAvh121* sequences and *PrAvh205* sequences, and may be descended from the ancestral gene for these two clades. The best current phylogeny for the genus shows *P. foliorum* to be the basal taxon in this group (Clade 8c), although with weak branch support [60]. Alternatively, the *PrAvh205* ortholog in *P. foliorum* has diverged from the other orthologs such that we were unable to amplify it using primers designed from the known sequences. The *PrAvh121* ortholog in *P. hibernalis* has a stop codon in the signal peptide and therefore is likely to be nonfunctional. As described in a previous publication [35], the *PrAvh121* alleles in the *P. ramorum* EU1 lineage are unique in that the first two-thirds of the alleles are nearly identical in nucleotide sequence while the last third are markedly diverged. One allele closely resembles the other *P. ramorum* alleles while the second allele more closely resembles the *P. lateralis* ortholog. The recently reported EU2 lineage is homozygous for this second allele [36]. Thus, *PrAvh121* exhibits transpecific polymorphism and recombination.

An additional member of this subfamily is *PrAvh36*, which is shorter than the other *P. ramorum* member genes by 39 or more bases and very different in sequence from the other genes in its last ~ 30 bases. It is difficult to determine the ancestry of this gene due to its divergence from the other subfamily members; however, phylogenetic analysis of the 5’ flanking region of the *PrAvh205* paralogs indicates that *PrAvh36* is also a paralog of *PrAvh205* (Figure 6B). Examining this gene relative to its paralogs in only a single lineage, one would think that this is a pseudogene. But unlike the other genes in this subfamily, there is no variation in *PrAvh36* among *P. ramorum* lineages, suggesting that this gene is, or was recently, subject to purifying selection.

We specifically tested for positive selection in the expansion of the *PrAvh205* clade, because this appeared to be the most dynamic group of genes in this subfamily. A recombination event was detected when alleles from all three *P. ramorum* clonal lineages were examined, the likely recombinant a *PrAvh60* allele in NA2. Therefore, we tested for selection using a nonrecombinant fragment that included the RXLR motif and three quarters of the C-terminal functional region. We found evidence for two categories of selection across branches using GAbranch with small sample AIC: dN/dS for the first class was 0.29 and for the second class 4.13. The assignment of branches to these two classes produced a 95% confidence set of 1491 models. The model averaged probability of dN>dS was greater than 0.90 for 9 branches, greater than 0.95 for 8 branches, and greater than 0.99 for 3 branches (Figure 6C). The branches leading to new genes (following an inferred duplication event) generally showed high probabilities of positive selection. There was also evidence for positive selection on the branches leading to certain alleles, for example the EU1 *PrAvh68* allele and within *PrAvh205*. When the recombinant *PrAvh60* allele was removed from the analysis (rather than truncated at the recombination breakpoint), results were very similar but model-averaged probabilities of dN>dS were slightly lower, such that the number of branches with probabilities greater than 0.90, 0.95, and 0.99 were 7, 4, and 2, respectively. This suggests a loss of power due to removal of the *PrAvh60* allele and not a qualitatively different outcome. Tests for site-specific selection found between one and three codons with significant positive selection (FEL method) or a high probability thereof (REL & M8a methods) (Table 2). Other codons showed moderate support for positive selection (0.05<*P*<0.1 or Pr>0.95). This subfamily does not contain the WY-domain.

**Table 2:** Positive selection on codons among *PrAvh205* orthologs and paralogs, for the nonrecombinant region from site 130 to 330.

	FEL		REL		M8a
Codon	dN-dS^a^	*P*	dN-dS^b^	Pr(dN>dS)	Pr(ω >1)^c^
45	*-8.37*	*0.02*	*-15.74*	*0.02*	**1.000** ^d^
58	0.9	0.09	0.94	0.98	0.954
67	1.04	**0.03**	0.99	**1.00**	**0.997**
69	1.20	0.10	0.91	**1.00**	**0.999**
91	0.87	0.06	0.96	0.98	0.977
96	0.79	0.09	0.94	0.98	0.955
98	0.32	0.70	0.68	0.98	0.982
108	0.61	0.30	0.81	0.94	0.969
110	0.97	0.24	0.82	**0.99**	0.986

^a^Normalized by dividing dN-dS by the total length of the tree.

^b^Posterior expectation of dN-dS. Rate classes can be found in Table S2.

^c^Probabilities from Bayes Empirical Bayes analysis. Model estimated ω was 5.39.

^d^This result appears to be a false positive as the other tests indicate significant negative selection on this codon.

**Figure 7:**
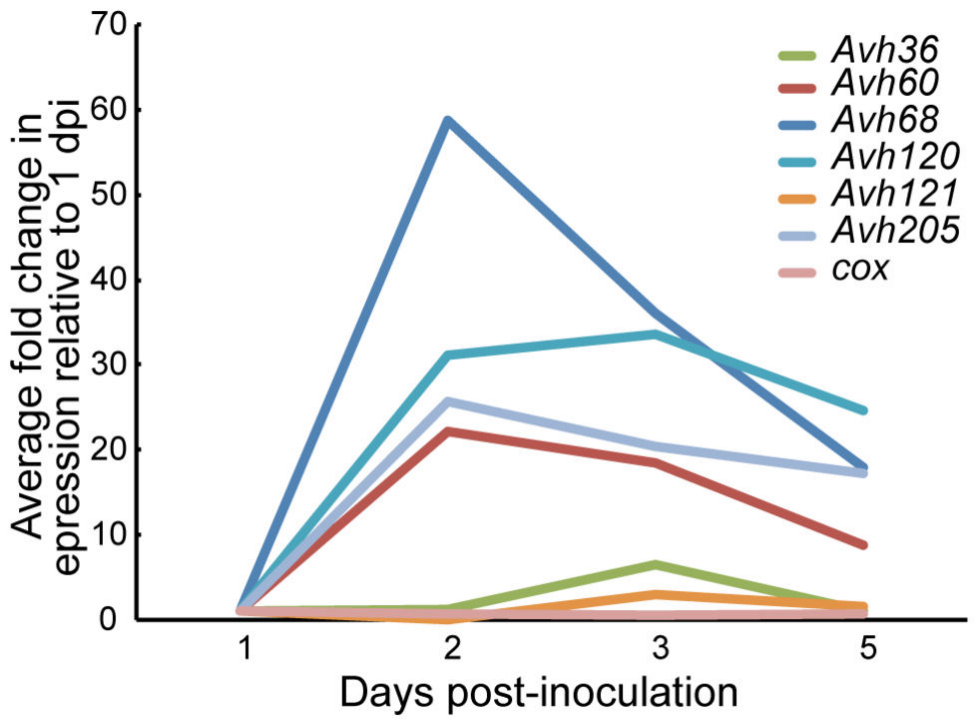
Expression of four RXLR effector genes in Rhododendron leaves infected with *P. ramorum*. In lineage NA1 isolate Pr-102, the genes *PrAvh60*, *PrAvh68*, *PrAvh120*, and *PrAvh205* exhibited induction of expression, on average, at 2, 3, and 5 days after inoculation relative to day 1. Expression of these genes was 20 to 60 times higher on day 2 relative to day 1. *PrAvh36* and *PrAvh121* were slightly induced on day 3. *PrAvh108* was not detected at any time point. The plant internal control (cox) was not induced relative to transcript levels on day 1.

 While there are homologs to this *P. ramorum* effector gene subfamily in *P. sojae* (*PsAvh226* and *PsAvh261*, [28]), these are not validated effectors. Therefore, we examined the expression of the *P. ramorum* genes in the host plant rhododendron to examine whether their patterns of expression were consistent with genes that are induced *in planta*. *PrAvh60*, *PrAvh68*, *PrAvh120*, and *PrAvh205* were on average strongly induced *in planta* at 22 to 58 times their level of expression one day post-inoculation (Figure 7). *PrAvh108* was not detected at any sampled time point, *PrAvh36* was marginally detected at days 2 and 3, and *PrAvh121* was detected only sporadically at low copy numbers. Analysis of variance (excluding only *PrAvh108*) indicated significant effects by gene (*P* < 0.0001), days post inoculation (*P* < 0.0001), and their interaction (*P* = 0.015). Analysis of variance by gene indicated significant induction for *PrAvh36*, *PrAvh60*, *PrAvh120*, and *PrAvh205*. Large variances in the observed copy numbers at each time point may be a consequence of the difficulty of working with rhododendron as a host. Given the apparent selection on these genes, we speculate that any loss of expression is evolutionarily recent. The selection detected in this subfamily is not likely to be unusual and we expect that many RXLR-class subfamilies would show similar patterns of diversification and selection. However, there is a limited number of subfamilies that do not also exhibit recombination and/or insertion/deletion polymorphisms, which makes testing for selection difficult.

## Conclusions

 Analysis of variation within RXLR-class effector gene subfamilies in *P. ramorum* and between *P. ramorum* and its closest known relatives highlights multiple evolutionary paths in the diversification of effector genes. We have observed loss or gain of repeated domains, recombination or gene conversion among paralogs, and selection on point mutations. Studies of effector gene function to date have focused on the effect of point mutations on key domains. It is not yet clear what effect exchange of domains through recombination would have on function and specificity of recombinant gene products. Recombination among paralogs has been shown to be pervasive in other gene families involved in coevolutionary interactions, including plant resistance genes [61,62], the malaria parasite *Plasmodium falciparum* virulence factor genes [63], and other classes of effector genes in oomycete and bacterial pathogens [11,59]. By sequencing genes in close relatives of *P. ramorum*, we uncovered repeated duplication events in *P. ramorum* since speciation from *P. lateralis*. Sequencing across the *P. ramorum* lineages revealed unexpected divergence of effector gene alleles. In particular, the EU1 lineage has unique recombinant *PrAvh17* alleles and a *P. lateralis*-like *PrAvh121* allele. Interestingly, in both these examples, the recombinant alleles diverge in the C-terminal functional region, which is the location of effector activity [20,30,64]. Sequencing the full genomes of isolates representing the *P. ramorum* lineages will reveal whether these distinguishing features of the EU1 lineage are pervasive throughout its effector gene repertoire. Furthermore, as functional characterization of RXLR-class effectors proceeds, we expect to better understand the functional and coevolutionary significance of the patterns of variation that we have observed in *P. ramorum*.

**Note.** Genome sequences for four *P. lateralis* isolates from Northern Ireland were published after this research was completed [65]. We conducted a blastn search of the genes we cloned from *P. lateralis* isolate 440 from Oregon against two of these genomes: MPF4 and SMSTG. These searches revealed allelic variation in the subfamily described in Figure 4 and an additional allele or gene in the subfamily analyzed in Figure 6. These results are presented in Figures S1 and S2.

## Supporting Information

Figure S1 **Maximum likelihood genealogy of the effector gene family in Figure 4 showing allelic variation in *P. lateralis*.** Alleles found in the *P. lateralis* MPF4 and SMSTG genomes are shown in bold. Whole coding sequences were used to construct the genealogy using the GTR substitution model. The branch lengths are drawn to scale and measured in the number of substitutions per site.
(TIF)

Figure S2 **Maximum likelihood genealogy of the effector gene family in Figure 6 with the additional sequence found in the *P. lateralis* genomes.** Alleles confirmed in the *P. lateralis* MPF4 and SMSTG genomes are shown in bold. Note the new *PrAvh205*-like allele (“P. lateralis new”). Whole coding sequences were used to construct the genealogy using the GTR substitution model. Branch support is indicated as a percentage of 500 bootstrap samples. The branch lengths are drawn to scale and measured in the number of substitutions per site.
(TIF)

File S1 **An Excel file containing the primers used for PCR and sequencing and for measuring expression of effectors.**
(XLSX)

File S2 **Nucleotide alignment of subfamily in Figure 1 in Fasta format.**
(FASTA)

File S3 **Nucleotide alignment of subfamily in Figure 2 in Fasta format.**
(FASTA)

Table S1 **GenBank accessions by species and locus.**
(PDF)

Table S2 **REL rate classes for *PrAvh205* orthologs and paralogs for the nonrecombinant region from site 130 to 330.**
(PDF)
