# Supplementary figures and images for: Evolution of RXLR-Class Effectors in the Oomycete Plant Pathogen Phytophthora ramorum
Source: PLoS One. 2013 Nov 7;8(11):e79347. doi: 10.1371/journal.pone.0079347 (PMC3820680; doi:10.1371/journal.pone.0079347)

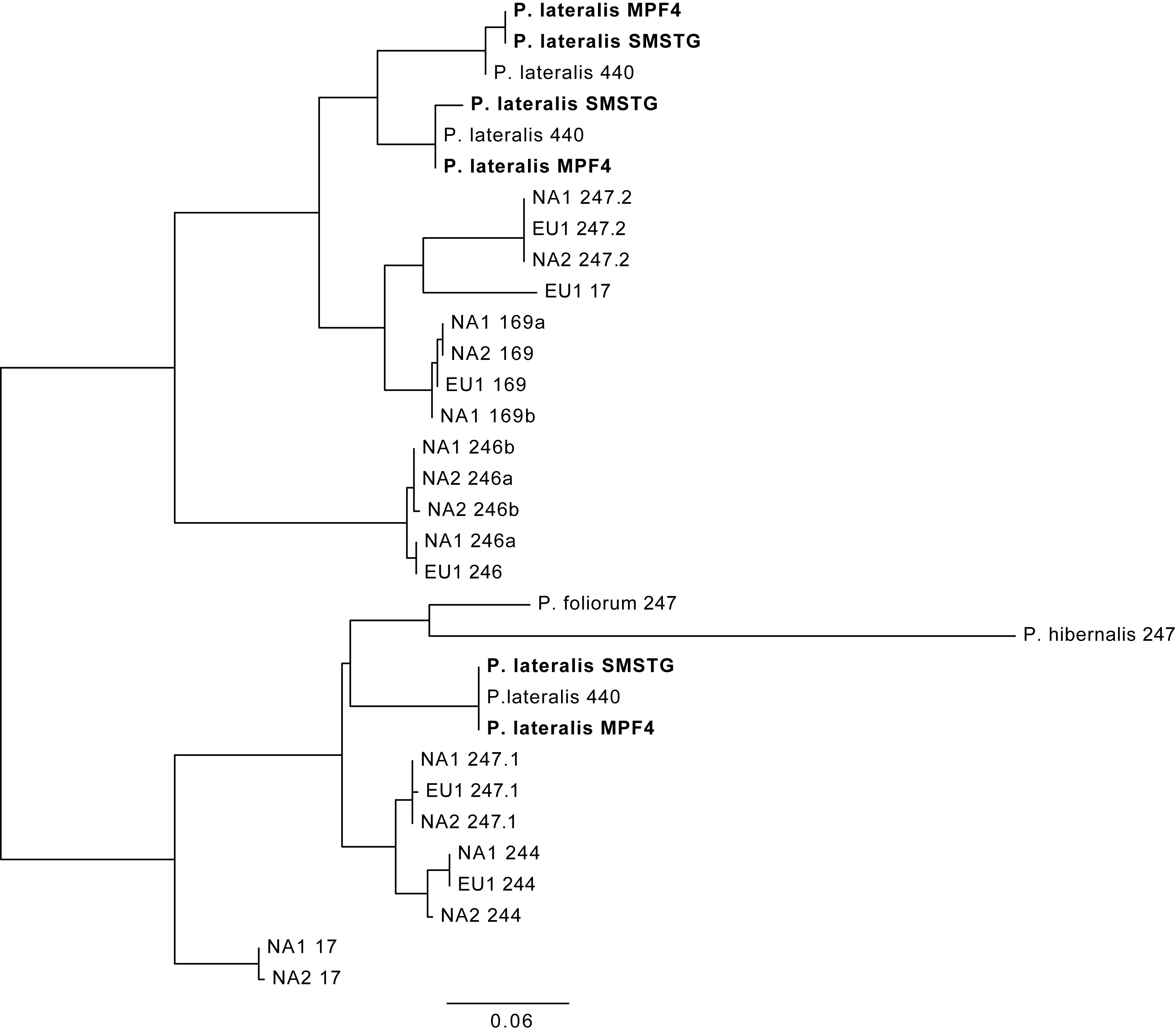

Supplement: Figure S1 — Maximum likelihood genealogy of the effector gene family in Figure 4 showing allelic variation in P. lateralis. Alleles found in the P. lateralis MPF4 and SMSTG genomes are shown in bold. Whole coding sequences were used to construct the genealogy using the GTR substitution model. The branch lengths are drawn to scale and measured in the number of substitutions per site. (TIF) [file pone.0079347.s001.tif]

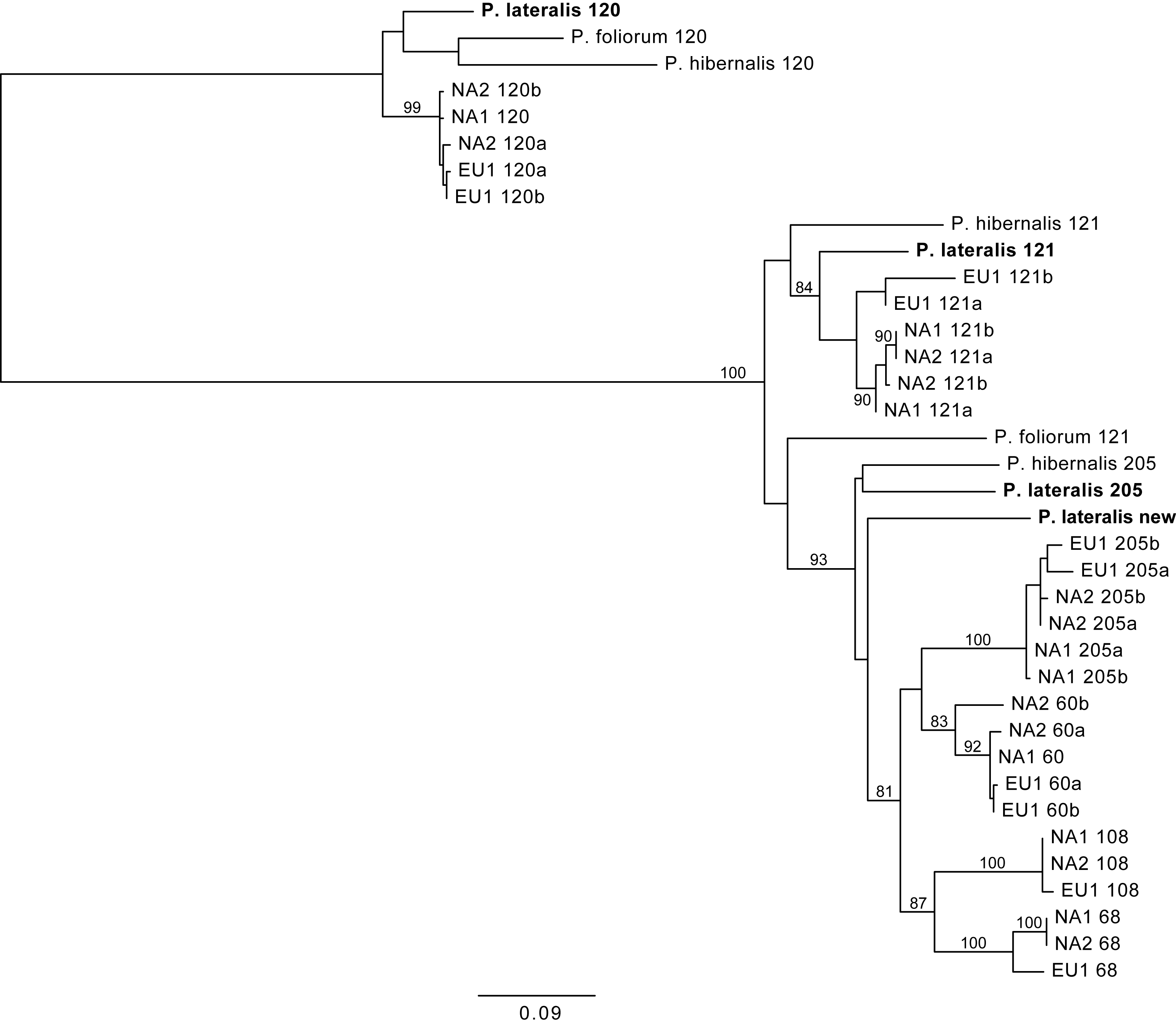

Supplement: Figure S2 — Maximum likelihood genealogy of the effector gene family in Figure 6 with the additional sequence found in the P. lateralis genomes. Alleles confirmed in the P. lateralis MPF4 and SMSTG genomes are shown in bold. Note the new PrAvh205-like allele (“P. lateralis new”). Whole coding sequences were used to construct the genealogy using the GTR substitution model. Branch support is indicated as a percentage of 500 bootstrap samples. The branch lengths are drawn to scale and measured in the number of substitutions per site. (TIF) [file pone.0079347.s002.tif]
