# Supplementary material for: Evolution of RXLR-Class Effectors in the Oomycete Plant Pathogen Phytophthora ramorum
Source: PLoS One. 2013 Nov 7;8(11):e79347. doi: 10.1371/journal.pone.0079347 (PMC3820680; doi:10.1371/journal.pone.0079347)
Supplement: Table S1 — GenBank accessions by species and locus. (PDF) [file pone.0079347.s006.pdf]

Table S1. GenBank accessions by species and locus.

Locus	Species	Genbank accession(s)
PrAvh17	*P. ramorum*	KF273355, KF273375, KF273363
PrAvh169/192	*P. ramorum*	KF273353, KF273354, KF273362, KF273374
PrAvh244	*P. ramorum*	KF273356, KF273364, KF273376
PrAvh246	*P. ramorum*	KF273357, KF273358, KF273365, KF273377, KF273378,
PrAvh247	*P. ramorum*	KF273359, KF273367, KF273379,
PrAvh247.2	*P. ramorum*	KF273360, KF273367, KF273368,
PrAvh169 homologs	*P. lateralis*	KF273371, KF273373
PrAvh247.1 homolog	*P. lateralis*	KF273372
PrAvh247.1 homolog	*P. hibernalis*	KF273370
PrAvh247.1 homolog	*P. foliorum*	KF273369
PrAvh36	*P. ramorum*	KF273350, KF273351, KF273352
PrAvh60	*P. ramorum*	KF273331- KF273335
PrAvh68	*P. ramorum*	KF273342- KF273344
PrAvh108	*P. ramorum*	KF273345- KF273347
PrAvh120	*P. ramorum*	EU850875-EU850895; EU850899-EU850902
PrAvh121[a]	*P. ramorum*	EU850903-EU850923; EU850927-EU850938
PrAvh205[a]	*P. ramorum*	KF273336-KF273341
PrAvh120 homolog[a]	*P. lateralis*	EU850896
PrAvh120 homolog[a]	*P. hibernalis*	EU850897
PrAvh120 homolog[a]	*P. foliorum*	EU850898
PrAvh121 homolog[a]	*P. lateralis*	EU850924
PrAvh121 homolog[a]	*P. hibernalis*	EU850925
PrAvh121/205 homolog[a]	*P. foliorum*	EU850926
PrAvh205 homolog	*P. lateralis*	KF273348
PrAvh205 homolog	*P. hibernalis*	KF273349

[a] Sequences were previously published in [35].
