# Supplementary material for: Evolution of RXLR-Class Effectors in the Oomycete Plant Pathogen Phytophthora ramorum
Source: PLoS One. 2013 Nov 7;8(11):e79347. doi: 10.1371/journal.pone.0079347 (PMC3820680; doi:10.1371/journal.pone.0079347)
Supplement: Table S2 — REL rate classes for PrAvh205 orthologs and paralogs for the nonrecombinant region from site 130 to 330. (PDF) [file pone.0079347.s007.pdf]

Table S2. REL rate classes for *PrAvh205* orthologs and paralogs for the nonrecombinant region from site 130 to 330.

Rate Class	1	2	3	4	5	6	7	8	9
dS	17.37	17.37	17.37	1.34	1.34	0.31	0.31	1.34	0.31
dN	0.17	0.29	1.38	0.17	0.29	0.17	0.29	1.38	1.38
dN-dS	-17.2	-17.08	-15.99	-1.17	-1.05	-0.14	-0.02	0.04	1.07
Prob.	0.014	0.005	0.008	0.119	0.041	0.387	0.133	0.069	0.225
